# Honor as Cultural Mindset: Activated Honor Mindset Affects Subsequent Judgment and Attention in Mindset-Congruent Ways

**DOI:** 10.3389/fpsyg.2016.01921

**Published:** 2016-12-09

**Authors:** Sheida Novin, Daphna Oyserman

**Affiliations:** ^1^Department of Psychology, Utrecht UniversityUtrecht, Netherlands; ^2^Institute for Social Research, University of Michigan, Ann ArborMI, USA; ^3^Department of Psychology, University of Southern California, Los AngelesCA, USA

**Keywords:** culture, situated cognition, lexical-decision, embodiment, gender

## Abstract

Honor values articulate gender roles, the importance of reputation in maintaining one’s place in society, and maintaining respect for the groups one belongs to. In that sense honor provides a template for organizing social interactions and hence may be functional even among people and societies that do not report valuing and endorsing honor. We test the prediction that honor influences judgment and attention when activated in two experiments (*N* = 538). Using a culture-as-situated cognition perspective, we predicted that activating one aspect of honor would activate other aspects, even among individuals who do not much endorse honor values. We tested these predictions among European Americans, a group that is not typically associated with honor values. In each study, participants were randomly assigned to experimental or control groups, which differed in one way: the experimental group read statements about honor values as a first step and the control group did not. Participants then judged stick-figure pairs (judging which is male; Study 1, *n* = 130) or made lexical decisions (judging whether a letter-string formed a correctly spelled word; Study 2, *n* = 408). In Study 1, experimental group participants were more likely to choose the visually agentic figure as male. In Study 2, experimental group participants were more accurate at noticing that the letter-string formed a word if the word was an honor-relevant word (e.g., noble), but they did not differ from the control group if the word was irrelevant to honor (e.g., happy). Participants in both studies were just above the neutral point in their endorsement of honor values. Individual differences in honor values endorsement did not moderate the effects of activating an honor mindset. Though honor is often described as if it is located in space, we did not find clear effects of where our letter strings were located on the computer screen. Our findings suggest a new way to consider how honor functions, even in societies in which honor is not a highly endorsed value.

## Introduction

Dueling, being a knight, and honor killings are each specific instances of honor behaviors that are specific to societies, times and places. Though different, each of these specific behaviors has a common intent—clarifying roles and exhibiting integrity to protect reputation and rank in a social unit. These elements of honor are quite consistent across time (historical analyses, Nisbett and Cohen, 1996; contemporary analyses, Rodriguez Mosquera et al., 2002a; Cross et al., 2013). In the current paper we take up an implication of this consistency, which is that individual and group-based marking of role, rank, and position within a social order may be rooted in functionally universal cultural themes of sustaining groups and relationships. Using this lens, honor behaviors highlight the need to invest in the group, to behave in ways that others in one’s in-group can rely on, and to preserve the group’s relative advantage. We propose honor to be part of a universally available knowledge network, or cultural mindset, that can be made accessible by subtle environmental cues. Cultural mindsets are knowledge networks that serve as meaning-making frameworks and influence what is attended to and which goals and mental procedures are salient (Oyserman, 2015, 2017).

Considering honor as a functionally universal cultural mindset implies that it is broadly available for use, rather than being used only in some societies, as previously assumed. Honor can be considered an element of moral reasoning (e.g., a binding value, see Graham et al., 2013). However, prior work has highlighted differences in how much social honor is valued and endorsed as compared to how much the dignity of each person within the group is valued and endorsed. Taking action to avenge slights and restore reputation is experienced as necessary by some people and in some societies more than others, the difference being whether third parties can be expected to intervene or if people and groups are obligated to act on their own. In ‘honor’ societies, what others’ think matters and vigilant attention to the possibility of losing face or losing respect is necessary (Stewart, 1994; Cohen et al., 1996; Margalit, 1996; Rodriguez Mosquera et al., 2002a; Gregg, 2007). In ‘dignity’ societies, others are de-emphasized and what matters is one’s own norms, values, and beliefs (Leung and Cohen, 2011; Cross et al., 2013). The implication typically drawn from these kinds of contrasts is that honor and dignity societies are quite different. Indeed the idea of societal difference is underscored by the fact that ‘honor’ and ‘dignity’ societies are located in different parts of the world – the Middle East, Mediterranean regions, Latin America, and Southern United States vs. Northern Europe and Northern United States.

Yet, this between-society difference does not necessarily imply that honor responses are only comprehensible in honor societies. Instead, it is possible that honor is a cultural mindset, an organized structure in memory, containing relevant content, procedures, and goals, even if it is not chronically activated. Whether an honor mindset is chronically activated or not and how much honor values are endorsed are interesting issues to be sure. However, neither of these interesting issues rules out the possibility that honor mindsets can be activated with resultant shift in judgment and attention toward honor relevant content, goals, and procedures even if honor is neither chronically activated nor particularly endorsed. In the current paper we test this cultural mindset activation prediction in two experiments with Northern American participants. In the next two sections we outline what honor is and then we use culture-as-situated cognition theory to explain what we mean by honor mindsets.

### The Concept of Honor

Honor is a multi-faceted and multi-level construct that includes the self (individual level), the family or other social unit (group level), and gender roles and norms (e.g., female chastity, male agency) as detailed next. Honor involves individual and group-based reputation for integrity, honesty, being true to one’s principles and marking place by earning respect, not tolerating disrespect and insults, and protecting oneself and one’s family, group or clan from face loss and reputational harm (e.g., Rodriguez Mosquera et al., 2002a,b; Gregg, 2005, 2007; Cross et al., 2013; Uskul et al., 2013; Novin et al., 2015). Beyond that, honor requires different things of men and women. Female honor involves shame, chastity, and purity (e.g., Rodriguez Mosquera et al., 2002a,b). Honorable women may gaze down as a sign of modesty (Vandello and Cohen, 2003). In contrast, male honor involves potency, including strength, power, and agentic action. Male honor is based on toughness, strength, and power to protect oneself, one’s property, and one’s family from insults and threats (Nisbett and Cohen, 1996; Vandello and Cohen, 2003). Honorable men stand up straight as a sign of confidence (e.g., IJzerman and Cohen, 2011).

Honor is also described as if it were an object, located in space. In English, honor is described as if it were ‘up’ on a vertical axis and on the ‘right’ on a horizontal axes. Thus, people can be worthy of *high* honors, *up*hold their honor, have a *high* sense of honor, can lose honor and sink down to being the *lowest* of the low so people will look *down* on them (see also Richardson et al., 2001 for the verticality of the word respect). Honorable deeds can be described as having done the *right* thing, restoring a situation to an honorable plan can be described as putting matters to *right*, and a person who is a trusted confidant can be described as being one’s *right* hand man.

The empirical honor literature contrasts ‘high-honor’ groups – Turks, Middle Easterners, Spaniards, Latin Americans, or Americans from the Southern United States with ‘dignity’ groups – Northern Europeans, or Americans from the North (e.g., Cohen et al., 1996; Rodriguez Mosquera et al., 2002a,b; Uskul et al., 2015).

Some between-group differences are dependent on experiencing threats to honor, with differences between ‘high-honor’ and ‘low-honor’ groups muted or absent when threats to honor are absent (Cohen and Nisbett, 1994; Beersma et al., 2003; IJzerman et al., 2007). Compared to samples from ‘dignity’ groups, samples from ‘high-honor’ groups perceive more conflict, feel more negative emotions, and act more defensively and aggressively (e.g., Cohen et al., 1996; IJzerman et al., 2007; Vandello et al., 2008; Barnes et al., 2012). One study showed a difference between high and low-honor groups in their response to shame, with ‘high-honor’ groups responding (e.g., via verbal disapproval) to protect their social image and ‘low-honor’ groups by simply withdrawing (Rodriguez Mosquera et al., 2008).

Other between-group differences are not dependent on experiencing threat. Individuals from high-honor societies are more involved in risk-taking (Barnes et al., 2012) and self-harm (Osterman and Brown, 2011), are more likely to engage in school-violence (Brown et al., 2009), and are less likely to seek mental health care (Brown et al., 2014). At the individual level, endorsement of honor values is associated with honor-relevant behavioral and emotional responses – for example defensive responses to personal or national threats (e.g., Barnes et al., 2014), especially among individuals from high-honor groups (e.g., Rodriguez Mosquera et al., 2002b; Uskul et al., 2015).

Taken together, evidence to date highlights the following: honor involves individual and group-based social reputation and ranking, specified masculine and feminine gender roles, and is often described as if it was physically located in space. Though typically studied as between group comparisons, we propose that these attributes may be functionally universal. Indeed, people describe honor as self-esteem, being respected by others, and moral behavior in both Northern American and Turkish groups (Cross et al., 2014). Some honor effects are found only if threat to honor occurs, others seem context-sensitive. In the next section, we build on these valuable findings, asking if honor might be considered a cultural mindset rather than being more part of some culture than others. To explain what we mean by a cultural mindset, we turn to the culture-as-situated cognition theory.

### Culture-as-Situated-Cognition

Culture-as-situated cognition theory has three core premises (Oyserman, 2015, 2017). The first premise is that human cognition is situated (Fiske, 1992) and contextualized (Schwarz, 2007; Smith and Semin, 2007). People do not act on all their available knowledge, but instead on that subset of their knowledge that is contextually activated and feels relevant at the moment of judgment. The second premise is that human culture developed from the survival necessity of connecting with others (Boyd and Richerson, 1985). The third premise is that culture is both a functional universal, found across societies, and a particular set of practices that together form a ‘good enough’ solution to the basic problems each society faces – sustaining the group over time, organizing relationships, and facilitating individual welfare (Schwartz, 1992; Cohen, 2001). Addressing these basic problems requires sensitivity to others’ perspectives and self-regulation so that one can connect, cooperate, and fit in, and motivation to initiate and invest in problem solving so that creative solutions can be generated (Oyserman, 2011, 2017).

From this social core, basic cultural mindsets develop that influence the meaning people make of their experiences. Cultural mindsets should function like other associative networks, the features of which are well known (e.g., Meier et al., 2012). For example, within associative networks, speed and accuracy of recognition are a function of prior experience with the same or related objects or words. Prior experience serves as a prime. When a previously encountered word or object is recognized, this is termed perceptual priming (e.g., Neely, 1991; Levy et al., 2004). When recognition is based on prior encounter with an associated word or object, rather than with the one currently presented, this is termed conceptual priming. That is, what was primed was a construct to which the word or object was related, rather than the word or object itself (e.g., Schacter and Buckner, 1998; Levy et al., 2004). Frequent or recent activation of a construct increases the likelihood that it will be used, influencing the accuracy and speed with which related constructs are recognized (e.g., Strack and Deutsch, 2004). What constitutes ‘related’ is a function of co-occurrence. If cultural mindsets function as other associative networks do, then encountering words and objects relevant to the mindset should increase accessibility of related words and objects.

The two most commonly studied cultural mindsets are individualistic and collectivistic mindsets. Both individualism and collectivism are related to the basic problems of survival. Individualism highlights individual welfare and reinforces innovation; collectivism emphasizes group boundaries and structuring relationships. Some societies emphasize individualism and some collectivism. Indeed, a large body of research demonstrates cross-societal variation in the chronic activation of these mindsets and in the specific practices associated with them (for reviews, Oyserman et al., 2002; Oyserman and Lee, 2008; Oyserman, 2011). At the same time, individualism and collectivism are both part of human culture and research shows that both individualistic and collectivistic mindsets are easily activated across different modern (meta-analysis Oyserman and Lee, 2008) and traditional societies (for examples Cronk, 2007; Cronk and Leech, 2012). Once activated, individualistic and collectivistic mindsets influence how ambiguous situations are perceived by influencing accessibility of an associative network of constructs (Oyserman, 2017).

We suggest that honor, though less studied, is also related to the basic problems of survival in the following ways. Honor highlights the need to invest in the group, the need to behave in ways that others in one’s in-group can rely on, and the need to preserve the group’s relative advantage. If honor is a cultural mindset, then people should have an available, though not necessarily activated, knowledge network of honor-related content, procedures, and goals. Just as individualistic and collectivistic cultural mindsets can be activated, it should be possible to activate an honor mindset with subtle contextual cues (Oyserman, 2011, 2017). Once activated, an honor mindset should serve as a meaning-making framework which influences affect, behavior, and cognition, including judgment and attention. Much in the way that stereotype threat effects are not dependent on endorsing stereotypes (Steele and Aronson, 1995), the influences of an honor mindset should be separate from how much honor values are endorsed. We looked to the literature for evidence that honor mindsets can be activated, finding a study that looked at effects on truth telling (Leung and Cohen, 2011) and a study that looked at effects on honor values (IJzerman and Cohen, 2011). As detailed next, these studies focused on honor values rather than on an activated honor mindset *per se*.

In the truth telling study, Leung and Cohen (2011) had participants watch video clips depicting violent retaliation to insult and assessed truth telling in an ostensibly unrelated task. Lying was lower in Latinos and white Southerners who agreed with what they saw in the videos compared to those who disagreed. The extent of agreement with the video had no effect on truth telling for non-Southerners. The implication is that watching violent retaliation to insult cues honor only if this particular aspect of honor is valued. In the honor values study, honor values were successfully cued in non-Southerners assigned to posture (upright not slouched) and word stem (honor not neutral) conditions before filling out an honor values scale (IJzerman and Cohen, 2011). While showing an effect on honor values, for a number of reasons this study cannot address our prediction that an activated honor mindset increases use of honor to make sense of the world. First, the dependent variable was endorsement of honor values, but we predict an effect separate from value endorsement. Second, effects required an embodied element, but whether embodiment is always needed is unclear. Third, IJzerman and Cohen do not specify whether the honor-relevant words in the word stem task (independent variable) were also in the honor scale (dependent variable). It is possible that effects are at least in part accounted for by the perceptual fluency of the repeated words. Repeated words would be easier to recognize and prior work shows that experienced ease can carry over to judgments of liking and truth (Schwarz et al., 2007).

As an example of the problem in interpreting IJzerman and Cohen’s study, participants in their experimental condition might have encountered the stem ho___ and filled in honor. Having seen the word honor before might make honor more *perceptually* fluent when encountered again on the values scale, resulting in higher endorsement of statements containing the fluent word if fluency is interpreted as truth. It is not clear that IJzerman and Cohen were interested in the distinction between perceptual and *conceptual* fluency but given our set of predictions, the underlying process matters. It is possible that subtle cues (i.e., completing an honor value scale) increase perceptual fluency (seeing a word once makes it easier to see it or agree with it when it is seen again soon after). However, to document that honor is a cultural mindset, we need to show that an activated honor mindset has effects that are due to conceptual fluency and not only due to perceptual fluency.

### The Current Studies

In two studies we tested the prediction that honor is a cultural mindset, a meaning-making lens that has downstream consequences for judgment and attention. We did so by testing differences in the judgments and perception of participants who either did or did not fill out an honor values scale prior to making judgment or reporting their perceptions. We predicted that an activated honor mindset would increase use of honor-relevant information in processing information for subsequent judgment and attention tasks, independently from how much honor values were explicitly endorsed. To test our prediction, we created an honor values scale based on Rodriguez Mosquera and colleagues’ (Rodriguez Mosquera et al., 2002a,b; Vandello and Cohen, 2003) commonly used honor values scale, which includes both individual-level and group-level statements. In the experimental condition participants read and responded to the honor values scale prior to the presentation of the dependent variable. We chose this method of activating a cultural mindset because it allowed us to rule out the alternative prediction, which is that effects would only be found for participants who endorsed honor cultural values (see Oyserman et al., 1998 for an example using individualism and collectivism values to activate these cultural mindsets). We created an honor values scale that omitted statements about gender norms and that included some honor relevant words but not others. These precautions allowed us to have a cultural mindset activation manipulation that was distinct from our dependent variables. This was necessary so that we could test our prediction of conceptual priming. Otherwise, it would have been possible that our effects were due to perceptual priming, as we argued might be the case for IJzerman and Cohen (2011). In Study 1 our method allowed us to test our prediction that honor mindsets include gender roles even though gender roles were not part of the activation task. In Study 2 our method allowed us to test our prediction that honor mindsets have both perceptual and conceptual consequences since some letter strings involved words relevant to honor that participants had not read in the mindset activation task. We used a longer version of the scale in Study 1 and a shorter version – a subset of the Study 1 scale, in Study 2.

Our dependent variable in Study 1 was a judgment task – judging which of two ambiguous figures was ‘male.’ In each pair, we used a different visual cue of potency taken from the gender literature. Male dominance and potency are associated with height and eye gaze (Campbell, 1996; Marsh et al., 2009). Similarly, larger mass (Ralls, 1976; Fallon and Rozin, 1985) and higher color contrast (e.g., Hogg, 1969; Prudica et al., 2007) are experienced as potent, dominant, and male, separate from whether this is factually true in the natural world. Therefore, we used these visual potency cues (color contrast, height, gaze, and body mass) by presenting pairs of figures that differed in each of these cues presented alone, and asking participants which figure was male.

Our dependent variable in Study 2 was also a judgment task – judging whether a string of letters presented on the screen was a correctly spelled word in English or not. Some of the words were irrelevant to honor, others were relevant to honor, and of these latter words, some were in the activation task and others were new. This allowed us to rule out a number of alternative possibilities. First, that effects might be found only among people who endorse honor values. Second, that effects might not be specific to honor –perhaps activating honor mindset increases motivation overall. Third, that effects might be only at the perceptual level –recognition of just previously seen words, but not at the conceptual level –recognition of new words that are conceptually associated with but not the same as previously presented words.

Finally, given linguistic evidence that honor is spatially represented, in Study 2 we added spatial location (top, to the right vs. bottom, to the left) to our design. Our goal was to examine the possibility that spatial location has a main or interactive effect – improving performance directly or in conjunction with activated honor mindset.

## Study 1

### Sample

Undergraduates [*N* = 130; *M*_age_ = 19.16, *SD* = 1.22; 44% male; 94.6% not from border South or Deep South as defined by Cohen and Nisbett (1994); 56.2% European American, 24.6% Asian American, 5.4% American other heritage, 4.6% International, 3.1% Hispanic American, 3.1% Arab American, 3.1% African American] fulfilled subject pool requirements by participating. Sample size was determined by our subject pool allocation. Allocation is set each semester by considering how many researchers ask for research participants and how many students sign up for research participation credit. Data were collected until subject pool enrollment was over. The study obtained IRB approval and participants granted their written informed consent.

### Procedure

**FIGURE 1:**
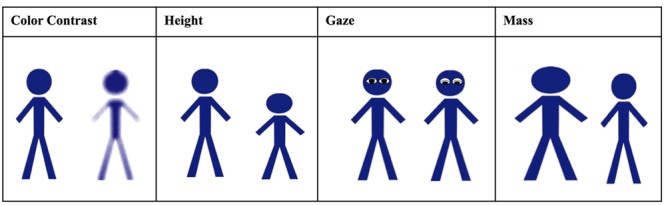
**Study 1: Figure pairs used.** Participants were asked to decide which one of the two figures in each pair represented the male character. In each pair, we have labeled the potency attribute – color contrast, height, gaze, and mass. This label was not presented to participants in the study itself.

Participants were seated in front of a computer terminal; instructions and randomization were automatized. Participants read and rated how much they agreed or disagreed (1 = *strongly disagree*, 7 = *strongly agree; M* = 5.01, *SD* = 0.58) with the 18 statements shown in **Table 1**. In the honor mindset activated condition (*n* = 66), the statements were about honor (e.g., “I prefer to live with honor, even if it means I will earn less money”). In the no cultural mindset activated condition (*n* = 64), the statements were not relevant to honor mindset (e.g., “I think breakfast is an important meal”). Next came the dependent variable, a visual task based on the task described by Semin and Palma (2014). The instructions “An artist wants to decide which of two figures to use to represent the male character in a story, click on your choice for the male character in each pair presented^1^,” preceded four pairs of ambiguous figures (**Figure 1**). In each pair visual potency differed so that one figure’s color contrast is sharper, one is taller, one has direct gaze, one has larger body mass than the other. We randomized figure position (right, left) and the order in which the pairs were presented.

**Table 1:** Study 1: Honor Values Scale and Filler Questionnaire Items.

Honor Values Scale (Used in Activated Honor Mindset Condition)
I prefer to live with honor, even if it means I will earn less money
To maintain my honor, I should not allow myself to be humiliated by others
Even if I lose social status if I still have my honor, I can respect myself
I would not disregard my honor even under tough life circumstances
My honor will likely be negatively affected if I do not attend to my family obligations
I would jeopardize the honor of my family if I behaved disgracefully
It is my duty to defend the honor of my family
It is important for me to keep face in front of others
I would lose face if others saw me misbehave
If I am embarrassed, I must not let it show or I will lose face
“My word is my bond” is how I feel; it would be dishonorable to behave otherwise
Honorable people do not cheat people who trust them
Loyalty is a core part of having honor
Acting right is necessary to maintain my honor
Reputation matters and should be vigorously defended
My honor depends to a high degree on the appreciation and respect of others
Disrespect damages honor
I would show I had no honor if I didn’t care “what others would say”

**Filler Questionnaire (Used in No Activated Mindset Condition)**

I think breakfast in an important meal
I like to eat a hot meal on a cold day
Eating a balanced diet should be easy to do
The saying “early to bed, early to rise, makes a person healthy, wealthy, and wise” is a good way to live my life
“Haste makes waste” makes no sense in consumer society
The saying “the early bird catches the worm” is reminder for me not to procrastinate
I like the fall season
Ice sparkling on tree branches brightens up even dark winter days
I’m outside a lot during the spring
Traffic can make me angry
I am in a better mood on sunny days
When packages are late I can get annoyed
I prefer well-organized classes
I dislike classes with lot of long readings
The college years should be pleasant
I can imagine taking up cooking as a hobby in the future
I like to read for pleasure
I enjoy working out

*7-point response scale (1 = *Strongly Disagree*, 7 = *Strongly Agree*). Honor values scale (α = 0.77, *M* = 5.01, *SD* = 0.58). The filler questions were meant to be of similar valence (*M* = 5.35, *SD* = 0.38) but not a scale (α = 0.46).*

### Analyses Plan

Each participant judged four pairs of figures, choosing which figure should be used to represent the male figure each time. Therefore, we used repeated measures logistic regression. Condition (activated honor mindset, no activated mindset), potency feature (height, color sharpness, gaze, and body mass), and participant gender were our independent variables. To test for possible group-level differences, we divided participants into those from groups previously identified in the literature as ‘honor’ groups and those previously identified in the literature as not from ‘honor’ groups. We did so by separating white, not from the Deep South participants, from all other participants (e.g., white participants from the Deep South, Hispanic, Asian, African American). For simplicity we label this as variable ‘region.’ Both region (Cohen and Nisbett, 1994; Stewart et al., 2006; IJzerman and Cohen, 2011; Leung and Cohen, 2011) and gender (Cihangir, 2013) have been associated with honor values in the literature. Therefore, we tested whether region or gender influenced judgment in our sample. Region did not influence judgment (*p*’s ≥ 0.280), but gender did –men were more likely to judge the taller figure as male (*p*_height_ = 0.047) and the sharper color contrast figure as male (*p*_contrast_ = 0.027). So gender, not region, was included in the final analyses presented next.

### Results and Discussion

**FIGURE 2:**
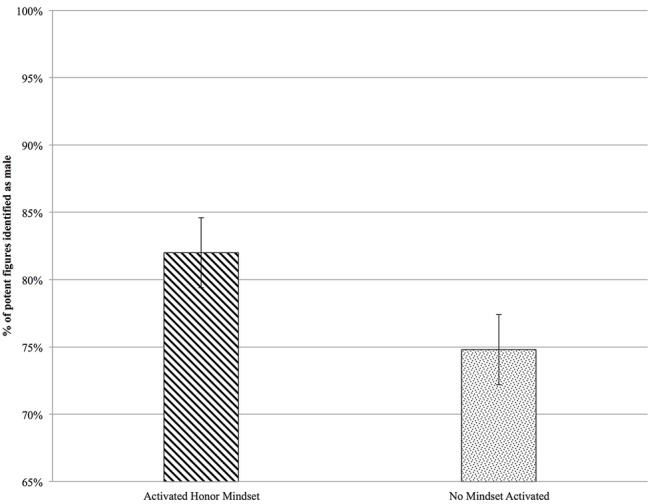
**Study 1: The effect of activating honor mindset on use of visual cues of potency to decide whether an ambiguous figure is male.** The activated honor mindset condition rated statements about honor prior to the visual task. The no activated mindset condition rated statements not about honor prior to the visual task. Error bars represent standard errors.

As predicted, activated honor mindset influenced judgment, as reflected in a main effect of mindset condition, *Wald X*^2^(1) = 6.41, *p* = 0.011, *w* = 0.22. This main effect is depicted graphically in **Figure 2**. Participants in the activated honor mindset condition (*M* = 81.53%, *SE* = 2.26%) were more likely to choose the more visually potent figure as male than were participants in the no activated mindset condition (*M* = 73.87%, *SE* = 2.28%). We also found a main effect of the specific visual potency feature used, *Wald X*^2^(3) = 103.22, *p* < 0.001, *w* = 0.89. Some of the specific visual potency features we used were more associated with maleness than others. However, honor mindset condition and potency feature did not interact, *Wald X*^2^(3) = 2.39, *p* = 0.495. This means that participants in the activated honor mindset condition were more likely to choose the more visually potent figure as the ‘male’ in each case, not just for some.

To test the prediction that the effect of an activated honor mindset was not a function of how much honor values were endorsed, we added the mean honor endorsement score to the regression equation. Since only participants in the activated honor mindset condition filled out the honor scale, only these participants were included in this analysis. As predicted, how much honor values were endorsed did not influence use of potency features, *Wald X*^2^(1) = 0.11, *p* = 0.740.

Study 1 results support our prediction that an activated honor mindset influences judgment separate from endorsing honor values in a sample of participants whose endorsement of honor values was moderate, just above the neutral point. Two strengths of Study 1 are first, the judgment task was subtle and so was unlikely to have demand characteristics and second, the honor scale omitted mention of gender so effects are unlikely to be due to the effect of being explicitly reminded of gender roles. Two limitations of Study 1, addressed in Study 2, are first, honor values were not assessed in the no activated cultural mindset group and second, we showed an effect of activated honor mindset on one aspect of honor – masculinity, but did not test whether activated honor mindset affected other aspects of honor. Therefore, in Study 2 we included a measure of honor values in the no mindset activated condition and an assessment of processing fluency – accuracy of recognizing honor words that were not included in the priming task, using words that cross the spectrum of individual, group, masculine and feminine components of honor.

## Study 2

### Sample

Undergraduates (*N* = 437; *M*_age_ = 18.84, *SD* = 1.68; 38% male; 91.5% right-handed) participated in a ‘word study’ as part of subject pool.^2^ Most (68.3%) were European American (14.5% Asian American, 5.7% African American, 5.5% International, 3.2% Hispanic American, 1.4% Arab American, 1.4% American other heritage). The study obtained IRB approval and participants granted their written informed consent.

### Procedure

Participants were seated in front of a computer terminal; instructions and randomization were automatized. Participants rated how much they agreed (1 = *strongly disagree*, 7 = *strongly agree; M* = 4.79, *SD* = 1.01) with five individual-level and group-level honor statements derived from the scale in Study 1 (**Table 2**; e.g., “My honor depends on the appreciation and the respect that others hold toward me”). They completed the honor scale either before (activated honor mindset condition) or after (no mindset activated condition) completing a lexical decision task. Handedness^3^, demographics, and understanding of instructions were obtained before thanking and debriefing participants. Participants (*n* = 29) who reported not understanding instructions were dropped from analyses, though key results do not change if they are included (see Supplementary Materials for Summary Tables for results); they did not differ from other participants in their demographics. ^4^

**Table 2:** Study 2: Honor Scale Item and Lexical Decision Task Letter Strings.

Honor Scale Items	Lexical Decision Task		
	Honor-irrelevant words	Honor-relevant words	Non-words
(1) My **honor** depends on the appreciation and the respect that others hold toward me. (2) To maintain my honor I should be loyal to my family, no matter what the circumstances are. (3) It is my duty to always **defend** the honor of my family. (4) To maintain my honor, I should always be prepared to defend my **reputation.** (5) To maintain my honor, I must not allow myself to be humiliated by others	Casual, Efficiency, Happy, Humor, Logical, Methodical, Miracle, Presents, Sympathetic, Talent	**Defend, Honor**, Noble, Prestige, Principles, Protect, Recognition, **Reputation, Respect**, Virtue	Accoptance, Actave, Acknuwledge, Autside, Emosational, Fergive, Fluwer, Inderstand, Laght, Leugh, Momories, Optomistic, Pasitive Prafit, Smole, Sniggle, Spirt, Twolight, Usoful, Woalthy

*Honor Scale *M* = 4.79, *SD* = 1.01, range 1.60 to 7.00, α = 0.73. Bolded words are the words that appeared in both the honor scale and in the lexical decision task.*

In the lexical decision task, participants saw a fixation point (+) presented in the middle of the screen for 200 ms followed by a letter-string. Their task was to report as quickly as they could without making a mistake if the string formed a correctly spelled word in English. To do so, participants were told to position their index fingers on the M key, labeled “word” and the V key, labeled “non-word.” The letter-string remained on the screen until the participant responded. All letter-strings were pronounceable in English. Twenty letter-strings did not form a correctly spelled word and 20 did and each letter-string was presented twice in randomized order for a total of 80 trials. Letter-strings were either above or below the fixation point (vertical axis) or to the right or left of the fixation point (horizontal axis). Participants were randomized to vertical or horizontal presentation. We manipulated whether the letter-strings forming honor words were located in positions that matched (up, right) or mismatched (down, left) the linguistic usage of honor. The statements and letters-strings are all shown in **Table 2**.

Of the 20 correctly spelled words, 10 were honor-relevant words (e.g., virtue) from our review of the honor literature that also came up in our pilot test (*N* = 101) in which participants were asked what comes to mind when thinking about honor. The other ten words had nothing to do with honor (e.g., talent), but were rated equally positively by a separate sample of students in a second pilot study (*N* = 37) using a 10-point scale (honor-relevant words *M* = 7.43, *SD* = 1.86, honor-irrelevant words *M* = 7.18, *SD* = 1.11, *p* = 0.104).

As can be seen in **Table 2**, six of the ten honor-relevant words were new, not presented in the honor values scale and four were old, presented in the honor values scale. We operationalized conceptual fluency as accuracy in recognizing the six not previously seen letter-strings as words and perceptual fluency as in recognizing the four previously seen letter-strings as words. We predicted that an activated honor mindset would increase both conceptual and perceptual fluency regardless how much honor values were endorsed.

### Analyses Plan

In the analyses below, we tested the hypothesized influence of accessible honor mindset on attention as operationalized as follows. First, we tested the effect of accessible honor mindset on response accuracy and latency in recognizing honor-relevant words in contrast to honor-irrelevant words. Second, we tested the effect of accessible honor mindset on accuracy and latency when spatial location matched (up, right) rather than mismatched (down, left) the linguistic usage of honor. Third, we tested the effect of accessible honor mindset as both a form of conceptual and a form of perceptual fluency. Fourth, we tested the effect of accessible honor mindset separate from level of honor values endorsement.

In order to test the first two operationalizations of the hypothesized influence of accessible honor mindset we used ANOVA’s with accuracy and latency as dependent variables and with Mindset Condition (activated honor mindset, no activated mindset), Word Type (honor-relevant words, honor-irrelevant words), Spatial Axis (up–down, left–right), and Match or Mismatch of Spatial Location to Honor (match, mismatch) as independent variables. There were two control variables. One control variable was accuracy in recognizing non-words because this controls both for general attention and for reading fluency. The other control variable was handedness given that the literature on response time indicates the influence of handedness (Casasanto, 2009), indeed, we found that right-handed participants faster than left-handed participants. As in Study 1 we tested for the possibility that participant gender and region influenced responses given that both have been associated with honor. Since neither participant demographic was associated with response, neither was included in the analyses.

To test the third operationalization of the hypothesized influence of accessible honor mindset we repeated the ANOVA analyses, but now Word Type consisted of honor-relevant words used in the scale (perceptual fluency), honor-relevant words not used in the scale (conceptual fluency), and honor-irrelevant words. To test the fourth and final operationalization of the hypothesized influence of accessible honor mindset we used regressions in order to add endorsement of honor values as a variable.

Assuming a speed-accuracy tradeoff, to improve accuracy, speed may need to be sacrificed and conversely, to improve speed, accuracy may need to be sacrificed (Dickman and Meyer, 1988). Given that instructions were to work as fast as one could without making mistakes, we expected an effect on accuracy (our primary dependent variable). The results of these analyses are presented below. For interested readers, effects on speed to accurate response are presented in the Supplementary Materials.

### Results and Discussion

Participants followed instructions and made few mistaken identifications of non-words as words or of words as non-words. These mistaken identification occurred in less than 10% of all responses (*M* = 7.4%, *SD* = 7.8%) and did not vary by condition, *t*(406) = 0.62, *p* = 0.534.

First, we contrasted honor-relevant and honor-irrelevant words (Supplementary Table S1 for full analysis) and found the predicted effect of activated honor mindset, as reflected in a significant two-way Word Type by Mindset condition interaction, *F*(1,397) = 11.62, *p* = 0.001, *d* = 0.34. Participants in the activated honor mindset condition were more accurate in recognizing letter-strings that formed honor-relevant words as words (*M* = 95.9%, *SE* = 0.5%) than participants in the no activated mindset condition (*M* = 93.7%, *SE* = 0.5%), *F*(1,403) = 9.09, *p* = 0.003, *d* = 0.30). Mindset condition did not influence accuracy in recognizing honor-irrelevant words, *F*(1,403) = 0.48, *p* = 0.489, *d* = 0.07, ruling out the possibility that filling out the honor scale increased motivation generally.

**FIGURE 3:**
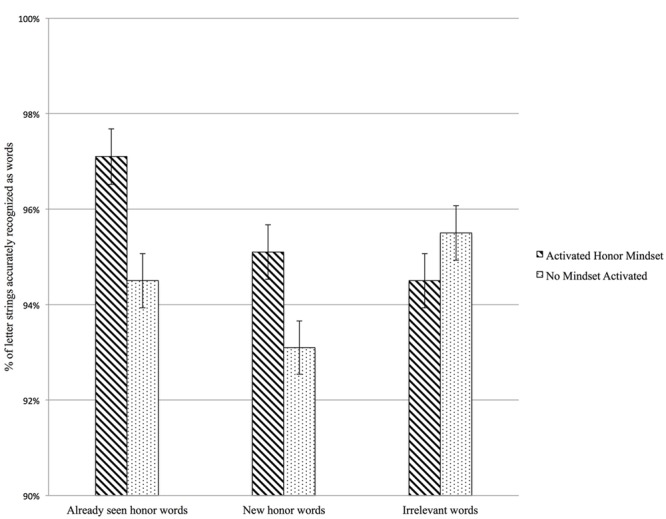
**Study 2: The effect of activating an honor mindset on percentage of letter-strings accurately recognized as words for honor-relevant words.** Already seen = words present in the honor scale, new = words not present in the honor scale, irrelevant = honor-irrelevant words. Error bars represent standard errors. Analyses include handedness and accuracy at recognizing non-words as controls.

Second, we contrasted already seen honor words, new honor words, and irrelevant to honor words (**Table 3**). As depicted graphically in **Figure 3**, the effect of activated honor mindset on recognizing honor-relevant words was found, regardless of whether participants had seen the words in the honor scale, *F*(1,403) = 7.03, *p* = 0.008, *d* = 0.26, or the words were new, *F*(1,403) = 5.87, *p* = 0.016, *d* = 0.24. These effects were not moderated by how much participants endorsed honor (*ps* > 0.339). As predicted, results indicate that an activated honor mindset facilitates accurate recognition of honor-relevant words, separate from endorsement of honor values.

**Table 3:** Study 2: Effect of Activated Mindset, Word Type, Spatial Axis and Spatial Match with Honor on Accuracy of Identifying Letter-Strings as Words for Honor Relevant Words (Presented in Scale and not Presented in Scale) and Honor Irrelevant Words.

	*df*	*F*	*d*	*p*
Main effects				
Word Type	2	14.06	0.53	<0.001
Mindset Condition	1	5.38	0.23	0.021
Spatial Axis	1	29.79	0.55	<0.001
Spatial Match	1	11.04	0.33	0.001
Interaction effects				
Mindset Condition × Spatial Match	1	0.61	0.08	0.434
Mindset Condition × Spatial Axis	1	2.46	0.16	0.117
Word Type × Mindset Condition	2	6.01	0.35	0.003
Spatial Match × Spatial Axis	1	12.82	0.36	<0.001
Word Type × Spatial Match	2	3.63	0.27	0.027
Word Type × Spatial Axis	2	15.14	0.55	<0.001
Mindset Condition × Spatial Match × Spatial Axis	1	9.92	0.32	0.002
Word Type × Mindset Condition × Spatial Match	2	1.30	0.16	0.275
Word Type × Mindset Condition × Spatial Axis	2	1.06	0.15	0.346
Word Type × Spatial Match × Spatial Axis	2	1.14	0.15	0.321
Word Type × Mindset Condition × Spatial Match × Spatial Axis	2	0.46	0.10	0.633
Controls				
Handedness	1	15.77	0.40	<0.001
Mean Accuracy Non-Words	1	461.80	2.16	<0.001
Error	397			

*Mindset Condition 1 = Activated Before, -1 = Not Activated, Assessed After lexical decision task; Spatial Match: 1 = Match to Honor Location (top or right), -1 = Mismatch to Honor Location (bottom or left); Spatial Axis: 1 = Vertical (above, below fixation point) -1 = Horizontal (right, left fixation point); Handedness: 1 = left-handed, -1 = right-handed = -1.*

Finally, we examined the effect of spatial location-honor concept match or mismatch. We conducted two analyses. First we contrasted honor-relevant and honor-irrelevant words (Supplementary Table S1) and then we contrasted already seen honor words, new honor words, and irrelevant to honor words (**Table 3**). Effects in both analyses were similar and did not fit the predicted effect of spatial location-honor concept match. Rather than the expected facilitation effect of honor words being spatially located to the top and right, analyses yielded complex and not easily interpretable location effects. Specifically, we found a two-way interaction of Word Type and Spatial Match (**Table 3**). *Post hoc* analyses revealed that participants were more accurate in recognizing honor-irrelevant words in the mismatch (down, left) than in the match (up, right) location, *F*(1,403) = 23.74, *p* < 0.001, *d* = 0.49. Accuracy at recognizing honor-relevant words did not differ as a function of location (seen before, *p* = 0.439; not seen before, *p* = 0.536, honor words). Endorsement of honor values did not moderate these effects (*p*s > 0.651). As **Table 3** details, we also found a Word Type by Spatial Axis interaction and a Spatial Match by Spatial Axis interaction, as well as a Mindset Condition by Spatial Match by Spatial Axis interaction. Follow-up analyses, however (see Supplementary Figure S1) did not provide interpretable insights, implying that activated honor mindset, as operationalized here, was not linked to spatial location in any clear way. Supplementary Materials (Supplementary Tables S2 and S3; Supplementary Figures S2 and S3) also provide analyses of speed to response, which show a similarly complex relationship to spatial match location.

Study 2 results support our predictions that honor is a cultural mindset that influences attention when activated, separately from how much honor is valued among participants who are not particularly high in honor value endorsement (scores were just above the neutral point). Compared to participants in the control condition, those in the honor mindset activation condition were more accurate in recognizing not only honor words they had seen before, but also new words relevant to the construct of honor that they had not seen before. Effects were specific to honor words; accuracy in recognizing other words did not differ between the groups. Effects were not moderated by how much participants endorsed honor words.

## General Discussion

Honor values articulate gender roles, the importance of reputation in maintaining one’s place in society, and maintaining respect for the groups one belongs to. In that sense, honor provides a template for organizing social interactions and hence may be functional even among people and societies that do not highly value and endorse honor. Culture-as-situated cognition theory predicts that contextual cues can activate honor mindsets, which include a network of associated constructs and ideas (e.g., male agency) and are used as a meaning-making lens even by individuals who do not much endorse honor values. We tested and found support for these predictions in two studies. In Study 1 experimental group participants were more likely to choose the visually agentic figure as male. In Study 2, experimental group participants were more accurate at noticing that the letter-string formed a word if the word was an honor relevant word (e.g., noble), but did not differ from the control group if the letter string formed a word that was irrelevant to honor (e.g., happy). In both studies participants’ mean valuation of honor was just above the neutral point and differences in their endorsement of honor values did not moderate the effect of activating an honor mindset. We also explored, but did not find, clear support for an effect of spatial location on accessibility of honor constructs. Our results have implications for research on honor and situated cognition, which we outline next.

### Implications

Honor research typically focuses on how much honor values are endorsed, honor-related attitudes, and behavior (e.g., Cohen et al., 1996; Rodriguez Mosquera et al., 2008) and on associations between endorsing honor-related values, attitudes, and behavior (e.g., Uskul et al., 2015). While studying endorsement of honor values is important, this focus on honor values does not test the effect of activating an honor mindset separate from endorsement of honor values. If our prediction is correct, then honor mindsets are knowledge structures, which influence judgment and attention when activated, separate from endorsement of honor values. Supporting our prediction, our results reveal an effect of activated honor mindset on judgment and attention, separate from endorsement of honor values. To our knowledge, these studies are the first to support the possibility that honor mindsets, like individualistic and collectivistic mindsets, are cultural mindsets, available in memory, whether or not chronically activated and whether or not honor values are endorsed. As such, honor is a plausible candidate as a functionally universal element of culture. From a culture-as-situated-cognition perspective, the universality of honor is likely given that honor involves a set of practices for regulating relationship (e.g., protecting), a core characteristic of what culture is. However, future studies need to test the functional universality of honor by examining whether an honor mindset can be activated in many different cultural groups.

Our results have everyday implications to the extent that honor mindsets are cued in everyday life. This does seem to be the case. For example, during the 2016 Republican Presidential Primary, one of the candidates engaged in critical comments about the physical appearance of another candidate’s spouse while at the same time hotly denying that his own hands were small or that the size of his hands somehow indicated that his penis size should be questioned. These comments only make sense in the context of honor mindset being cued with the implication that failing to preserve one’s spouse from criticism and attacks on one’s own physical endowment must be responded to.

Our results also have implications for situated cognition research. We show effects of construct activation separate from construct endorsement. The other area of situated cognition in which the separate effects of construct activation and endorsement have been studied is the domain of stereotyping (Wheeler and Petty, 2001). Researchers have documented that stereotypes that are ‘in the air,’ by which is meant that when they are culturally available for use, they influence judgment, perception, and behavior, even among participants who do not endorse the stereotype (Steele and Aronson, 1995). This is true for everyone; whether or not they are members of the stereotyped group. It only matters if the stereotypes activated – made accessible for use in the moment (Wheeler and Petty, 2001). We show the same effect for honor, implying that honor is a construct that is available for use, even if not endorsed.

### Limitations and Directions for Future Research

Of course no set of studies is without limitations nor can it can rule out all alternative explanations and a number of limitations and alternative explanations to our findings should be considered. First, in Study 1 we tested effects for cues of maleness, not femaleness. Second, in Study 2 we tested effects for honor as up and not for dishonor is down. Third, in both studies we used lab settings and participants in the U.S. who were mostly non-Southern European Americans. Fourth, in both studies we used an “active” control group in which control participants read and rated filler items rather than a “no-prime” control. We address each of these issues next.

First, in Study 1 we found that activating an honor mindset increased use of visual potency as a cue of maleness. We did not test for the effect of honor mindset on use of visual information as cues of femaleness. While not undermining our current finding, it is possible that activating an honor mindset also influences perception of femaleness. Consider purity and chastity as an element of an honor representation of femaleness, Schnall (2014) argues that a basic metaphor for purity and chastity is a vessel. This implies that an activated honor mindset should also increase use of vessels and closed-containers as cues of femaleness. For example, when an honor mindset is activated, people may be more likely to judge cups as more female than plates, or chests of drawers as more female than tables. This possibility fits our current results and warrants testing in future research.

Second, prior research has shown that positivity is associated with the vertical axis – positive is up (e.g., Meier and Robinson, 2004). Hence, a possible alternative explanation for our findings in Study 2 is that our results are due to the positivity of our honor words. This seems an unlikely alternative explanation of our results for two reasons: first, we chose honor-relevant and honor-irrelevant words that had been rated as equally positive in our pretest, and second, our honor priming effects were shown only for honor-relevant and not for honor-irrelevant words. However, it does imply an important next step for future research. Future research could include both (positive) honor words and (negative) dishonor words to test for effects in both directions. Including positive and negative words would allow us to test if the reason for the current weak effects of location is because honor is located both at the top (honor) and at the bottom (dishonor) of the vertical axis (see also Xie and Zhang, 2014). Including positive and negative words in a within subjects design would allow a more subtle test of spatial location effects because people are more sensitive to change than to fixed position.

Together, both our studies focused on the effect of an honor mindset on specific indices of cognition: attention and judgment. This can be considered as an initial step into the examination of the consequences of an honor mindset on cognition beyond the existing work on honor in relation to behavioral and emotional responses to an honor-threatening situation. Future studies are needed to systematically examine the causal processes of an honor mindset on cognitive procedures (how people think) and mental content (what people think), as well as on affect and behavior.

Third, our studies are lab-based and use mostly non-Southern European American participants. Using a laboratory procedure allowed us to show effects on use of visual cues and to isolate conceptual effects of an activated honor mindset and to document that our understanding of location cues is currently limited. This came at the cost of ecological validity. Future research should seek out ways to test the consequences of an activated honor mindset in more ecologically valid ways. For example, future research could look at available materials such as media representations to see if honor cues are used to market products that do not seem to be related to honor. The more honor cues are used for marketing, the more likely it is that honor will be chronically cued separate from whether honor is actually valued. For example, does an activated honor mindset increase willingness to pay for products that fit male potency and female purity? Furthermore, our effects are shown in non-Southern white participants. Although this is an important first step because this is a group in which effects are not expected, cross-cultural replications are needed to test the theory that honor is indeed a universal cultural mindset. Moreover, given that honor values are more salient in some cultural groups (e.g., Turkish people), it would be theoretically worthwhile to examine whether the endorsement of honor values influences judgment and attention in these typical honor groups.

Fourth, our studies used an active control group in which control participants read and rated filler statements rather than a no-prime control group. Having an active control means that both groups had first read and considered their perspective on the same number of statements prior to engaging in the dependent variable task. However, this means that we cannot know what might be a natural state of affairs. No-prime control groups might be able to tell us about that but at the same time, would be non-parallel to the primed group because they had something on the mind prior to the dependent variable task and that itself might have created as dissimilarity. Future research could consider various other ways of creating either control groups or alternatively of contrasting honor and other cultural mindsets (individualistic and collectivistic) to further clarify effects.

Taken together, our studies suggest a new way of considering honor, as a cultural mindset, rather than as a between-group or individual difference variable. Using this formulation allowed us to document effects of an activated cultural mindset on perception separate from endorsement of honor values. It allowed us to show that effects of honor mindsets are dependent on them coming to mind. Future research is needed to understand when honor is likely to be experienced as relevant when it is activated.

## Ethics Statement

Study 1: Health Sciences and Behavioral Sciences Institutional Review Board (University of Michigan, Ann Arbor) IRB EXEMPTION STATUS: The IRB HSBS has reviewed the study referenced above and determined that, as currently described, it is exempt from ongoing IRB review, per the following federal exemption category: EXEMPTION #2 of the 45 CFR 46.101.(b): Research involving the use of educational tests (cognitive, diagnostic, aptitude, achievement), survey procedures, interview procedures or observation of public behavior. Consent (computerized): You are being asked to participate voluntarily and anonymously for half an hour’s subject pool credit. You will be presented with a questionnaire and a perception task. The study should take less than half an hour but you will receive the full half hour credit. You can stop your participation at any time, without giving a reason. By clicking the arrow below you provide your consent to participate in the study. Debriefing form (computerized): Thank you for participating in our study. You were asked to complete a questionnaire and to identify which of two stick figures was male. Everyone did the stick figure task, but some people completed an honor questionnaire first and some a bogus questionnaire. We want to examine if completing an honor questionnaire first will influence how people judge which stick figure is male. For more information about research in this area you can go to these references (IJzerman and Cohen, 2011; Leung and Cohen, 2011). Study 2: Health Sciences and Behavioral Sciences Institutional Review Board (University of Michigan, Ann Arbor) IRB EXEMPTION STATUS: The IRB HSBS has reviewed the study referenced above and determined that, as currently described, it is exempt from ongoing IRB review, per the following federal exemption category: EXEMPTION #2 of the 45 CFR 46.101.(b): Research involving the use of educational tests (cognitive, diagnostic, aptitude, achievement), survey procedures, interview procedures or observation of public behavior. Consent form (computerized): You are being asked to participate voluntarily and anonymously for half an hour’s subject pool credit in the Word study. You will see strings of letters and as quickly as possible say if you are seeing a word or a non-word (nonsense letters). The study should take less than half an hour but you will receive the full half hour credit. You can stop your participation at any time, without giving a reason. By clicking the arrow below you provide your consent to participate in the study. Debriefing form (computerized): Thank you for participating in our study. You were asked to identify whether each string of letters represented a word or a non-word and to fill out a questionnaire. Some people did the word task first and filled out the questionnaire first. We want to examine if words that are related to honor are quicker recognized if they are presented in a specific spatial place (left, right, up, down). The questionnaire focused on honor values in daily life, which might influence reaction time. For more information about this research in this area, you can go to these references (Schubert, 2005; Schubert and Semin, 2010). Thank you very much for participating!

## Author Contributions

SN and DO contributed to the design of the study, literature search, initial draft of the manuscript, analyses, and revisions of the work. SN and DO gave approval for the final version of the manuscript and agree to be accountable for all aspects of the work.

## Conflict of Interest Statement

The authors declare that the research was conducted in the absence of any commercial or financial relationships that could be construed as a potential conflict of interest.

## Footnotes

1 Participants were not told what the other figure would be, so they might have concluded that the other figure was female or less male or had neither gender.

2 We used a larger sample size than Study 1 because we added the spatial location factor and effect size was not clear. Sample size each semester was limited by our allocation in University of Michigan’s subject pool. The study was run in two sets (one in fall semester, one in winter semester). We requested a prescreening question to parallel Cohen and Nisbett (1994) and obtain the state participants had grown up if they grew up in the U.S., obtaining IRB approval for this. However, the question was erroneously omitted from the prescreening process leaving us without this information at the individual level. So we obtained university-level distributions, finding that less than 5% of undergraduates at the University of Michigan (4.8%) come from border South or Deep South as defined by Cohen and Nisbett (1994). It is unlikely that our effects were driven by this small subgroup.

3 Handedness influences mental representations of spatial location (Casasanto, 2009).

4 *M*_age_ = 18.86, *SD* = 1.71; 38.5% male; 68.9% European American, 14.7% Asian American; 5.1% African American, 5.1% International students, 3.2% Hispanic American, 1.5% Arab American, 1.5% American other heritage; 91.7% right-handed.
