# Supplementary material for: Honor as Cultural Mindset: Activated Honor Mindset Affects Subsequent Judgment and Attention in Mindset-Congruent Ways
Source: Front Psychol. 2016 Dec 9;7:1921. doi: 10.3389/fpsyg.2016.01921 (PMC5145876; doi:10.3389/fpsyg.2016.01921)
Supplement: Supplementary file 1 [file Table_1.DOCX]

**Supplemental Materials**

We present two sets of analyses, one set with participants who reported understanding instructions (Tables S1 to S3, *n*= 408) and the other set with all participants, regardless of whether they said they understood instructions (Tables S4 to S7, N = 437).

Effects on accuracy are presented in two ways, comparing accuracy at recognizing as words letter strings composing honor-relevant vs. honor-irrelevant words (Tables S1, S4) and comparing accuracy at recognizing as words letter-strings composing new honor-relevant words, already seen honor-relevant words, or honor-irrelevant words (Table S5).

Effects on speed are presented in comparing speed at correctly recognizing as words letter-strings composing honor-relevant vs. honor-irrelevant words (Tables S2, S6) and comparing speed at accurately recognizing as words letter-strings composing new honor-relevant words, already seen honor-relevant words, or honor-irrelevant words (Tables S3, S7).

Relevant Figures are also presented.

Table S1.

*Study 2:* *Effect of Activated Mindset, Word Type, Spatial Axis and Spatial Match With Honor on Accuracy of Identifying Letter-Strings as Words for Honor-Irrelevant and Honor-Relevant Words*

|  | *df* | *F* | *d* | *p* |
| --- | --- | --- | --- | --- |
| *Main effects* |  |  |  |  |
| Word Type | 1 | 26.31 | 0.51 | <.001 |
| Mindset Condition | 1 | 2.37 | 0.15 | .125 |
| Spatial Axis | 1 | 18.49 | 0.43 | <.001 |
| Spatial Match | 1 | 18.03 | 0.43 | <.001 |
| *Interaction effects* |  |  |  |  |
| Mindset Condition X Spatial Match | 1 | 1.67 | 0.13 | .197 |
| Mindset Condition X Spatial Axis | 1 | 4.32 | 0.21 | .038 |
| Word Type X Mindset Condition | 1 | 11.62 | 0.34 | .001 |
| Spatial Match X Spatial Axis | 1 | 17.73 | 0.42 | <.001 |
| Word Type X Spatial Match | 1 | 7.26 | 0.27 | .007 |
| Word Type X Spatial Axis | 1 | 26.71 | 0.52 | <.001 |
| Mindset Condition X Spatial Match X Spatial Axis | 1 | 10.14 | 0.32 | .002 |
| Word Type X Mindset Condition X Spatial Match | 1 | 2.44 | 0.16 | .119 |
| Word Type X Mindset Condition X Spatial Axis | 1 | 2.11 | 0.15 | .147 |
| Word Type X Spatial Match X Spatial Axis | 1 | 0.57 | 0.08 | .450 |
| Word Type X Mindset Condition X Spatial Match X Spatial Axis | 1 | 0.12 | 0.03 | .734 |
| *Controls* |  |  |  |  |
| Handedness | 1 | 10.32 | 0.32 | .001 |
| Mean accuracy non-words | 1 | 472.82 | 2.18 | <.001 |
| Error | 397 |  |  |  |

*Note*. Mindset Condition 1=Activated Before, -1=Not Activated, Assessed After lexical decision task; Spatial Match: 1=Match to Honor Location (top or right), -1=Mismatch to Honor Location (bottom or left); Spatial Axis: 1= Vertical (above, below fixation point) -1= Horizontal (right, left fixation point); Handedness: 1= left-handed, -1= right-handed = -1
