# Supplementary material for: Honor as Cultural Mindset: Activated Honor Mindset Affects Subsequent Judgment and Attention in Mindset-Congruent Ways
Source: Front Psychol. 2016 Dec 9;7:1921. doi: 10.3389/fpsyg.2016.01921 (PMC5145876; doi:10.3389/fpsyg.2016.01921)
Supplement: Supplementary file 2 [file Table_2.DOCX]

Table S2.

*Study 2:* *Effect of Activated Mindset, Word Type, Spatial Axis and Spatial Match With Honor on Speed in Accurately Identifying Letter-Strings As Words for Honor-Irrelevant and Honor-Relevant Words*

|  | *df* | *F* | *d* | *p* |
| --- | --- | --- | --- | --- |
| *Main effects* |  |  |  |  |
| Word Type | 1 | 1.17 | 0.11 | .280 |
| Mindset Condition | 1 | 0.01 | 0.01 | .929 |
| Spatial Axis | 1 | 2.04 | 0.14 | .154 |
| Spatial Match | 1 | 2.17 | 0.15 | .141 |
| *Interaction effects* |  |  |  |  |
| Mindset Condition X Spatial Match | 1 | 0.08 | 0.03 | .782 |
| Mindset Condition X Spatial Axis | 1 | 0.79 | 0.09 | .374 |
| Word Type X Mindset Condition | 1 | 6.58 | 0.26 | .011 |
| Spatial Match X Spatial Axis | 1 | 0.17 | 0.04 | .679 |
| Word Type X Spatial Match | 1 | 66.55 | 0.82 | <.001 |
| Word Type X Spatial Axis | 1 | 0.00 | 0.00 | .947 |
| Mindset Condition X Spatial Match X Spatial Axis | 1 | 0.01 | 0.01 | .906 |
| Word Type X Mindset Condition X Spatial Match | 1 | 1.85 | 0.14 | .174 |
| Word Type X Mindset Condition X Spatial Axis | 1 | 1.12 | 0.11 | .290 |
| Word Type X Spatial Match X Spatial Axis | 1 | 6.26 | 0.25 | .013 |
| Word Type X Mindset Condition X Spatial Match X Spatial Axis | 1 | 0.12 | 0.04 | .729 |
| *Controls* |  |  |  |  |
| Handedness | 1 | 0.67 | 0.08 | .415 |
| Mean speed non-words | 1 | 993.52 | 3.17 | <.001 |
| Error | 396 |  |  |  |

*Note*: Mindset Condition 1=Activated Before, -1=Not Activated, Assessed After lexical decision task; Spatial Match: 1=Match to Honor Location (top or right), -1=Mismatch to Honor Location (bottom or left); Spatial Axis: 1= Vertical (above, below fixation point) -1= Horizontal (right, left fixation point); Handedness: 1= left-handed, -1= right-handed = -1
