# Supplementary material for: Honor as Cultural Mindset: Activated Honor Mindset Affects Subsequent Judgment and Attention in Mindset-Congruent Ways
Source: Front Psychol. 2016 Dec 9;7:1921. doi: 10.3389/fpsyg.2016.01921 (PMC5145876; doi:10.3389/fpsyg.2016.01921)
Supplement: Supplementary file 3 [file Table_3.DOCX]

Table S3.

*Study 2:* *Effect of Activated Mindset, Word Type, Spatial Axis and Spatial Match With Honor on Speed in Accurately Identifying Letter-Strings As Words for Honor-Relevant Words (Already Seen= Words Present in the Honor Scale, New= Words Not Present in the Honor Scale, Irrelevant= Honor-Irrelevant)*

|  | *df* | *F* | *d* | *p* |
| --- | --- | --- | --- | --- |
| *Main effects* |  |  |  |  |
| Word Type | 2 | 2.06 | 0.20 | .128 |
| Mindset Condition | 1 | 0.47 | 0.07 | .496 |
| Spatial Axis | 1 | 2.47 | 0.16 | .117 |
| Spatial Match | 1 | 0.00 | 0.00 | .983 |
| *Interaction effects* |  |  |  |  |
| Mindset Condition X Spatial Match | 1 | 0.07 | 0.03 | .935 |
| Mindset Condition X Spatial Axis | 1 | 1.39 | 0.12 | .239 |
| Word Type X Mindset Condition | 2 | 5.55 | 0.34 | .004 |
| Spatial Match X Spatial Axis | 1 | 0.44 | 0.07 | .510 |
| Word Type X Spatial Match | 2 | 32.96 | 0.82 | <.001 |
| Word Type X Spatial Axis | 2 | 0.49 | 0.10 | .615 |
| Mindset Condition X Spatial Match X Spatial Axis | 1 | 0.08 | 0.03 | .781 |
| Word Type X Mindset Condition X Spatial Match | 2 | 0.97 | 0.14 | .380 |
| Word Type X Mindset Condition X Spatial Axis | 2 | 1.02 | 0.14 | .360 |
| Word Type X Spatial Match X Spatial Axis | 2 | 3.44 | 0.27 | .033 |
| Word X Mindset Condition X Spatial Match X Spatial Axis | 2 | 0.17 | 0.06 | .840 |
| *Controls* |  |  |  |  |
| Handedness | 1 | 1.81 | 0.19 | .179 |
| Mean speed non-words | 1 | 848.13 | 4.14 | <.001 |
| Error | 395 |  |  |  |

*Note*: Mindset Condition 1=Activated Before, -1=Not Activated, Assessed After lexical decision task; Spatial Match: 1=Match to Honor Location (top or right), -1=Mismatch to Honor Location (bottom or left); Spatial Axis: 1= Vertical (above, below fixation point) -1= Horizontal (right, left fixation point); Handedness: 1= left-handed, -1= right-handed = -1
