# Supplementary material for: Honor as Cultural Mindset: Activated Honor Mindset Affects Subsequent Judgment and Attention in Mindset-Congruent Ways
Source: Front Psychol. 2016 Dec 9;7:1921. doi: 10.3389/fpsyg.2016.01921 (PMC5145876; doi:10.3389/fpsyg.2016.01921)
Supplement: Supplementary file 4 [file Table_4.DOCX]

Table S4.

*Study 2:* *Effect of Activated Mindset, Word Type, Spatial Axis and Spatial Match With Honor on Accuracy of Identifying Letter-Strings As Words for Honor-Irrelevant and Honor-Relevant Words*

|  | *df* | *F* | *d* | *p* |
| --- | --- | --- | --- | --- |
| *Main effects* |  |  |  |  |
| Word Type | 1 | 32.07 | 0.55 | <.001 |
| Mindset Condition | 1 | 0.28 | 0.05 | .600 |
| Spatial Axis | 1 | 15.91 | 0.39 | <.001 |
| Spatial Match | 1 | 7.68 | 0.27 | .061 |
| *Interaction effects* |  |  |  |  |
| Mindset Condition X Spatial Match | 1 | 3.47 | 0.18 | .063 |
| Mindset Condition X Spatial Axis | 1 | 6.91 | 0.26 | .009 |
| Word Type X Mindset Condition | 1 | 8.04 | 0.28 | .005 |
| Spatial Match X Spatial Axis | 1 | 7.93 | 0.27 | .005 |
| Word Type X Spatial Match | 1 | 9.40 | 0.30 | .002 |
| Word Type X Spatial Axis | 1 | 29.17 | 0.52 | <.001 |
| Mindset Condition X Spatial Match X Spatial Axis | 1 | 12.98 | 0.35 | <.001 |
| Word Type X Mindset Condition X Spatial Match | 1 | 2.06 | 0.14 | .152 |
| Word Type X Mindset Condition X Spatial Axis | 1 | 3.15 | 0.17 | .077 |
| Word Type X Spatial Match X Spatial Axis | 1 | 0.16 | 0.04 | .694 |
| Word Type X Mindset Condition X Spatial Match X Spatial Axis | 1 | 0.64 | 0.08 | .424 |
| *Controls* |  |  |  |  |
| Handedness | 1 | 11.41 | 0.33 | .001 |
| Mean accuracy non-words | 1 | 357.60 | 1.84 | <.001 |
| Error | 424 |  |  |  |

*Note*: Mindset Condition 1=Activated Before, -1=Not Activated, Assessed After lexical decision task; Spatial Match: 1=Match to Honor Location (top or right), -1=Mismatch to Honor Location (bottom or left); Spatial Axis: 1= Vertical (above, below fixation point) -1= Horizontal (right, left fixation point); Handedness: 1= left-handed, -1= right-handed = -1
