# Supplementary material for: Honor as Cultural Mindset: Activated Honor Mindset Affects Subsequent Judgment and Attention in Mindset-Congruent Ways
Source: Front Psychol. 2016 Dec 9;7:1921. doi: 10.3389/fpsyg.2016.01921 (PMC5145876; doi:10.3389/fpsyg.2016.01921)
Supplement: Supplementary file 5 [file Table_5.DOCX]

Table S5.

*Study 2:* *Effect of Activated Mindset, Word Type, Spatial Axis and Spatial Match With Honor on Accuracy of Identifying Letter-Strings As Words for Honor-Relevant Words (Already Seen= Words Present in the Honor Scale, New= Words Not Present in the Honor Scale, Irrelevant= Honor-Irrelevant)*

|  | *df* | *F* | *d* | *p* |
| --- | --- | --- | --- | --- |
| *Main effects* |  |  |  |  |
| Word Type | 2 | 16.13 | 0.55 | <.001 |
| Mindset Condition | 1 | 1.34 | 0.11 | .248 |
| Spatial Axis | 1 | 25.74 | 0.49 | <.001 |
| Spatial Match | 1 | 3.28 | 0.18 | .071 |
| *Interaction effects* |  |  |  |  |
| Mindset Condition X Spatial Match | 1 | 0.21 | 0.04 | .192 |
| Mindset Condition X Spatial Axis | 1 | 0.48 | 0.07 | .051 |
| Word Type X Mindset Condition | 2 | 4.03 | 0.28 | .019 |
| Spatial Match X Spatial Axis | 1 | 0.21 | 0.04 | .192 |
| Word Type X Spatial Match | 2 | 4.69 | 0.30 | .010 |
| Word Type X Spatial Axis | 2 | 15.91 | 15.91 | <.001 |
| Mindset Condition X Spatial Match X Spatial Axis | 1 | 12.43 | 0.34 | <.001 |
| Word Type X Mindset Condition X Spatial Match | 2 | 1.20 | 0.15 | .304 |
| Word Type X Mindset Condition X Spatial Axis | 2 | 1.60 | 0.17 | .203 |
| Word Type X Spatial Match X Spatial Axis | 2 | 0.96 | 0.13 | .385 |
| Word Type X Mindset Condition X Spatial Match X Spatial Axis | 2 | 0.43 | 0.09 | .652 |
| *Controls* |  |  |  |  |
| Handedness | 1 | 15.38 | 0.38 | <.001 |
| Mean accuracy non-words | 1 | 343.13 | 1.80 | <.001 |
| Error | 423 |  |  |  |

*Note*: Mindset Condition 1=Activated Before, -1=Not Activated, Assessed After lexical decision task; Spatial Match: 1=Match to Honor Location (top or right), -1=Mismatch to Honor Location (bottom or left); Spatial Axis: 1= Vertical (above, below fixation point) -1= Horizontal (right, left fixation point); Handedness: 1= left-handed, -1= right-handed = -1
