# Supplementary material for: Honor as Cultural Mindset: Activated Honor Mindset Affects Subsequent Judgment and Attention in Mindset-Congruent Ways
Source: Front Psychol. 2016 Dec 9;7:1921. doi: 10.3389/fpsyg.2016.01921 (PMC5145876; doi:10.3389/fpsyg.2016.01921)
Supplement: Supplementary file 6 [file Table_6.DOCX]

Table S6.

*Study 2:* *Effect of Activated Mindset, Word Type, Spatial Axis and Spatial Match With Honor on Speed in Accurately Identifying Letter-Strings As Words for Honor-Irrelevant and Honor-Relevant Words*

|  | *df* | *F* | *d* | *p* |
| --- | --- | --- | --- | --- |
| *Main effects* |  |  |  |  |
| Word Type | 1 | 0.56 | 0.07 | .454 |
| Mindset Condition | 1 | 0.03 | 0.02 | .959 |
| Spatial Axis | 1 | 1.74 | 0.13 | .188 |
| Spatial Match | 1 | 2.52 | 0.15 | .113 |
| *Interaction effects* |  |  |  |  |
| Mindset Condition X Spatial Match | 1 | 0.04 | 0.02 | .841 |
| Mindset Condition X Spatial Axis | 1 | 0.98 | 0.10 | .324 |
| Word Type X Mindset Condition | 1 | 9.56 | 0.30 | .002 |
| Spatial Match X Spatial Axis | 1 | 0.10 | 0.03 | .747 |
| Word Type X Spatial Match | 1 | 69.84 | 0.81 | <.001 |
| Word Type X Spatial Axis | 1 | 0.27 | 0.05 | .607 |
| Mindset Condition X Spatial Match X Spatial Axis | 1 | 0.00 | 0.00 | .997 |
| Word Type X Mindset Condition X Spatial Match | 1 | 1.99 | 0.14 | .159 |
| Word Type X Mindset Condition X Spatial Axis | 1 | 0.03 | 0.02 | .857 |
| Word Type X Spatial Match X Spatial Axi2 | 1 | 4.74 | 0.21 | .030 |
| Word Type X Mindset Condition X Spatial Match X Spatial Axis | 1 | 0.00 | 0.00 | .997 |
| *Controls* |  |  |  |  |
| Handedness | 1 | 0.31 | 0.05 | .579 |
| Mean speed non-words | 1 | 1129.19 | 3.27 | <.001 |
| Error | 423 |  |  |  |

*Note*: Mindset Condition 1=Activated Before, -1=Not Activated, Assessed After lexical decision task; Spatial Match: 1=Match to Honor Location (top or right), -1=Mismatch to Honor Location (bottom or left); Spatial Axis: 1= Vertical (above, below fixation point) -1= Horizontal (right, left fixation point); Handedness: 1= left-handed, -1= right-handed = -1
