# Supplementary material for: Honor as Cultural Mindset: Activated Honor Mindset Affects Subsequent Judgment and Attention in Mindset-Congruent Ways
Source: Front Psychol. 2016 Dec 9;7:1921. doi: 10.3389/fpsyg.2016.01921 (PMC5145876; doi:10.3389/fpsyg.2016.01921)
Supplement: Supplementary file 7 [file Table_7.DOCX]

Table S7.

*Study 2:* *Effect of Activated Mindset, Word Type, Spatial Axis and Spatial Match With Honor on Speed in Accurately Identifying Letter-Strings As Words for Honor-Relevant Words (Already Seen= Words Present in the Honor Scale, New= Words Not Present in the Honor Scale, Irrelevant= Honor-Irrelevant)*

|  | *df* | *F* | *d* | *p* |
| --- | --- | --- | --- | --- |
| *Main effects* |  |  |  |  |
| Word Type | 2 | 0.90 | 0.13 | .408 |
| Mindset Condition | 1 | 0.52 | 0.07 | .471 |
| Spatial Axis | 1 | 2.27 | 0.15 | .133 |
| Spatial Match | 1 | 0.00 | 0.00 | .994 |
| *Interaction effects* |  |  |  |  |
| Mindset Condition X Spatial Match | 1 | 0.11 | 0.03 | .997 |
| Mindset Condition X Spatial Axis | 1 | 1.07 | 0.10 | .302 |
| Word Type X Mindset Condition | 2 | 7.33 | 0.37 | .001 |
| Spatial Match X Spatial Axis | 1 | 0.19 | 0.04 | .661 |
| Word Type X Spatial Match | 2 | 34.24 | 0.81 | <.001 |
| Word Type X Spatial Axis | 2 | 0.17 | 0.06 | .846 |
| Mindset Condition X Spatial Match X Spatial Axis | 1 | 0.03 | 0.02 | .867 |
| Word Type X Mindset Condition X Spatial Match | 2 | 1.05 | 0.14 | .351 |
| Word Type X Mindset Condition X Spatial Axis | 2 | 0.29 | 0.07 | .750 |
| Word Type X Spatial Match X Spatial Axis | 2 | 3.71 | 0.27 | .025 |
| Word X Mindset Condition X Spatial Match X Spatial Axis | 2 | 0.18 | 0.06 | .839 |
| *Controls* |  |  |  |  |
| Handedness | 1 | 1.07 | 0.10 | .302 |
| Mean speed non-words | 1 | 903.81 | 2.93 | <.001 |
| Error | 421 |  |  |  |

*Note*: Mindset Condition 1=Activated Before, -1=Not Activated, Assessed After lexical decision task; Spatial Match: 1=Match to Honor Location (top or right), -1=Mismatch to Honor Location (bottom or left); Spatial Axis: 1= Vertical (above, below fixation point) -1= Horizontal (right, left fixation point);
