# Supplementary figures and images for: Honor as Cultural Mindset: Activated Honor Mindset Affects Subsequent Judgment and Attention in Mindset-Congruent Ways
Source: Front Psychol. 2016 Dec 9;7:1921. doi: 10.3389/fpsyg.2016.01921 (PMC5145876; doi:10.3389/fpsyg.2016.01921)

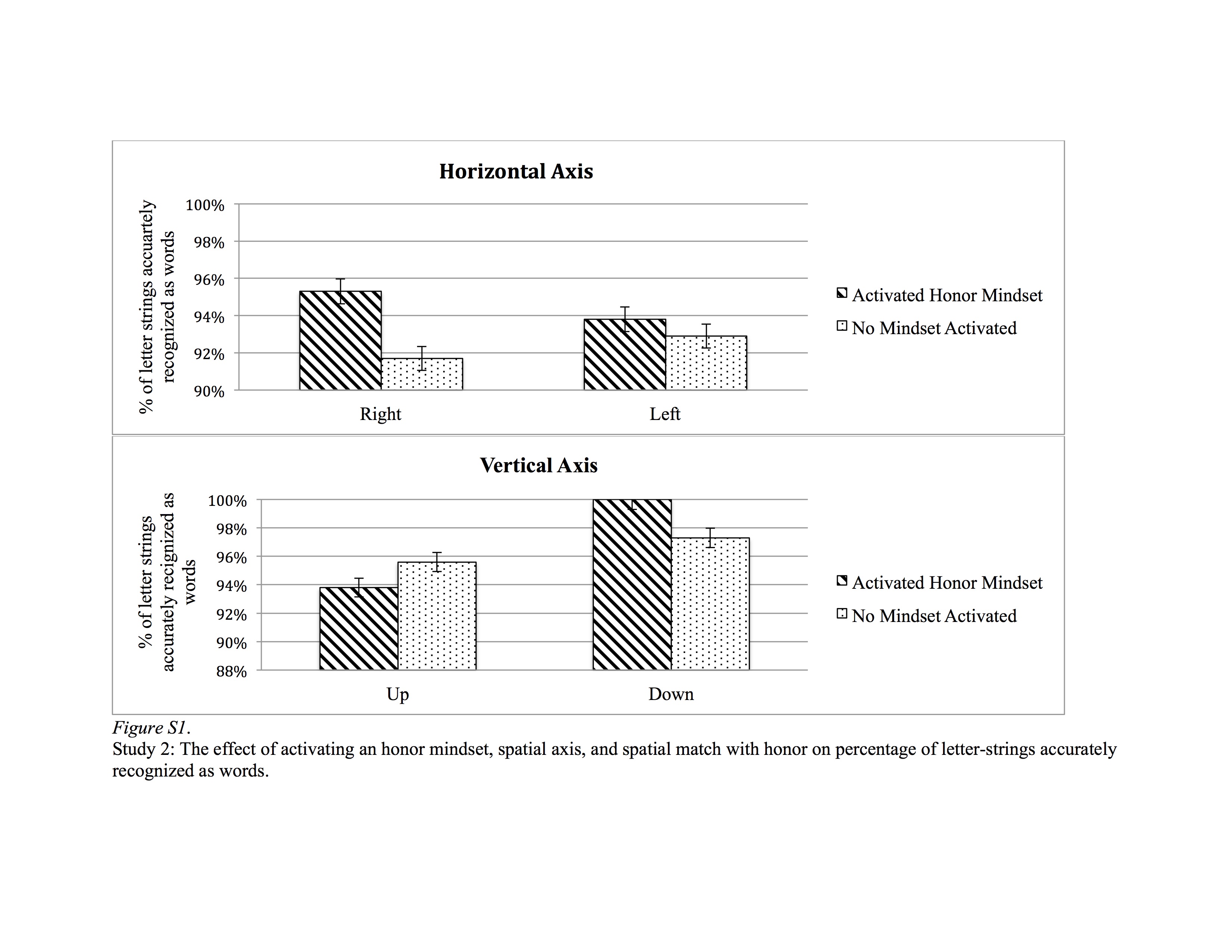

Supplement: Supplementary file 8 [file Image_1.JPEG]

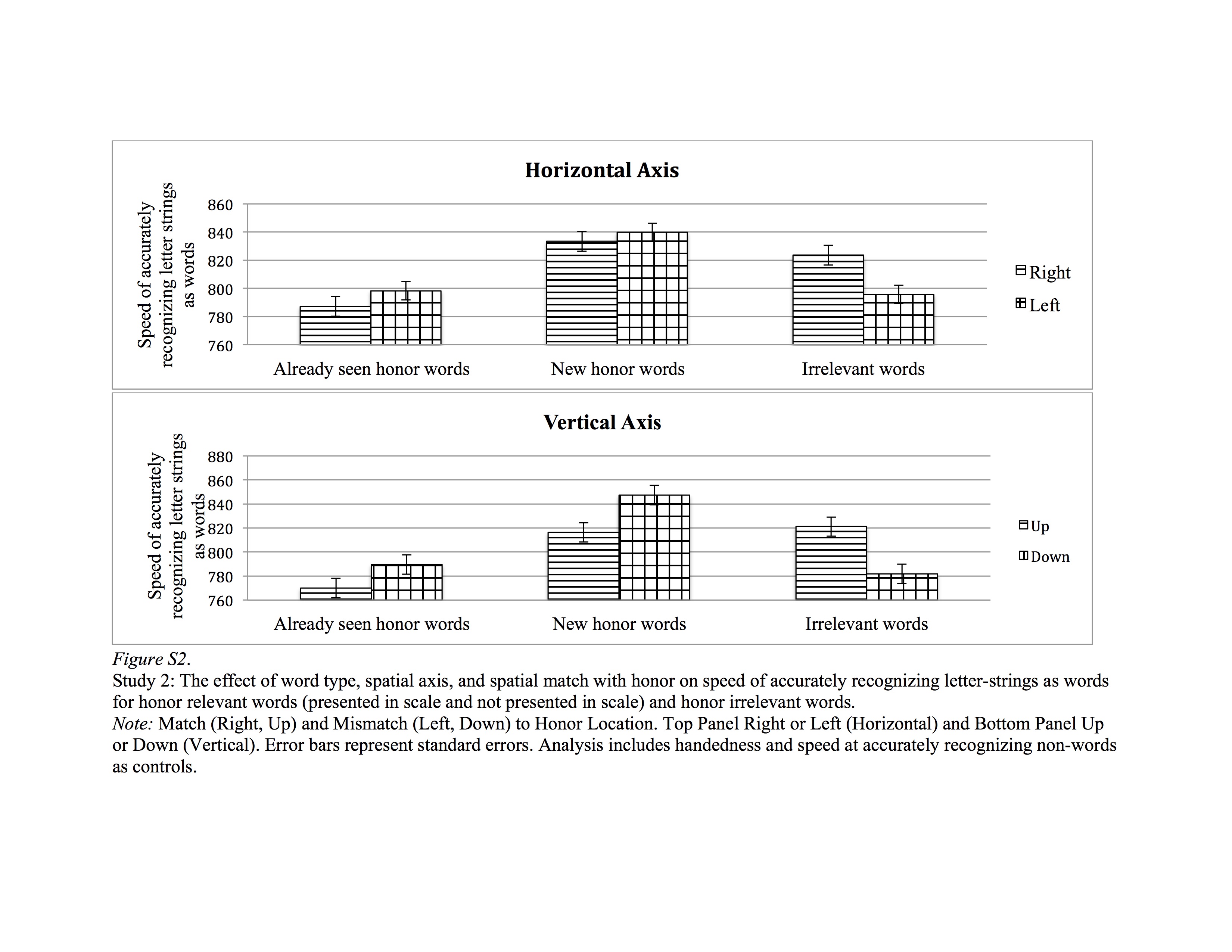

Supplement: Supplementary file 9 [file Image_2.JPEG]

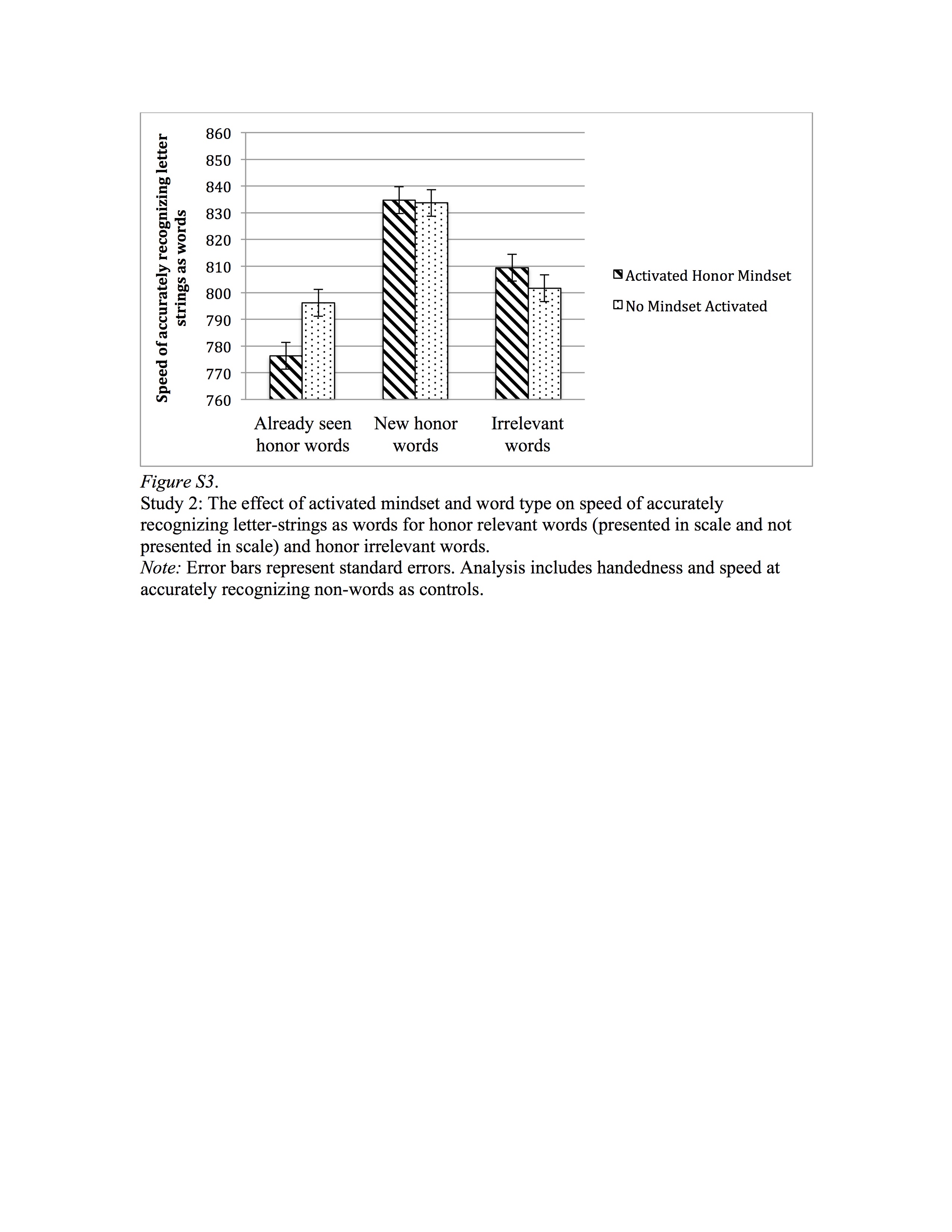

Supplement: Supplementary file 10 [file Image_3.JPEG]
